# IMPLEMENTATION OF ENGAGE IN A GERIATRIC MENTAL HEALTH SERVICE LINE WITH NONSPECIALIST PROVIDERS

**DOI:** 10.1093/geroni/igae098.3414

**Published:** 2024-12-31

**Authors:** Matthew Schurr, Emily Post, Teresa Walker, Brenna Renn

**Affiliations:** University of Nevada Las Vegas, Las Vegas, Nevada, United States; University of Nevada Las Vegas, Las Vegas, Nevada, United States; University of Nevada Las Vegas, Las Vegas, Nevada, United States; University of Nevada Las Vegas, Las Vegas, Nevada, United States

## Abstract

Despite development of efficacious psychosocial treatments for depression in older adults, implementation in real-world settings is limited, particularly in the context of patient comorbidities and across interprofessional teams. Engage is an empirically-supported psychotherapy for late-life depression but has yet to translate from randomized controlled trials to widespread implementation. Engage’s utility as a brief, structured behavioral protocol holds potential for feasible dissemination across multiple provider disciplines. This study evaluated a real-world training and pilot implementation of Engage psychotherapy across a geriatric mental health care hospital team. Care managers (N = 22) across interprofessional healthcare backgrounds (occupational therapy, social work, nursing) participated in training, consultation, and implementation of Engage with older adult patients with depression. Upon completion of the implementation trial, providers participated in a 1-hour focus group to provide feedback about their experience. Focus group interviews were double-coded using thematic analysis to extract implementation themes informed by the Consolidated Framework for Implementation Research (CFIR). Thirteen themes emerged related to CFIR constructs of innovation adaptability, innovation design, critical incidents, compatibility, access to knowledge and information, need, and capability. Overall findings suggest that Engage is a feasible treatment method that fits the needs of providers, patients, and larger health care systems. Barriers to implementation included depressive symptom burden, patient complexity, and provider concerns related to self-efficacy and previous related experiences. Implementation trials and elicitation of provider feedback to inform commensurate modifications should be a staple procedure to ensure optimal implementation of newly adopted mental health treatment services.

